# β-Cyclodextrin and Oligoarginine Peptide-Based Dendrimer-Entrapped Gold Nanoparticles for Improving Drug Delivery to the Inner Ear

**DOI:** 10.3389/fbioe.2022.844177

**Published:** 2022-04-11

**Authors:** Jia Luo, XueXin Lin, LiLing Li, JingQian Tan, Peng Li

**Affiliations:** ^1^ Department of Otolaryngology Head and Neck Surgery, The Third Affiliated Hospital of Sun Yat-Sen University, Guangzhou, China; ^2^ Department of Otolaryngology Head and Neck Surgery, The Eighth Affiliated Hospital of Sun Yat-Sen University, Shenzhen, China

**Keywords:** drug delivery, dexamethasone, inner ear, spiral ganglion, outer hair cells, nerve fibers

## Abstract

Here, we developed a safe and highly effective nanocarrier using β-cyclodextrin (β-CD) and oligoarginine peptide (Arg8)-modified dendrimer-entrapped gold nanoparticles (Au@CD-PAMAM-Arg8), with a diameter of 5 nm, for improved delivery of dexamethasone (Dex) to the inner ear. The properties and *in vivo* distribution of the Au@CD-PAMAM-Arg8 were assessed *in vitro*, and a streptomycin (SM) ototoxicity model was used *in vivo*. Flow cytometry analysis of HEIOC1 cells treated with Au@CD-PAMAM-Arg8 and Au @CD-PAMAM at different time intervals indicated that cell uptake efficiency of the drug delivery carrier Au@CD-PAMAM-Arg8 was higher than that of Au @CD-PAMAM. Au@CD-PAMAM-Arg8 carrying Dex (Au@CD-PAMAM-Arg8/Dex) were mainly distributed in hair cells, the spiral ganglion, lateral wall, and nerve fibers and had stronger protective effects on the inner ear than Dex administration alone. *In vivo* tracer tests revealed that tympanic injection was significantly more effective than posterior ear injection, muscle injection, and tail vein injection, whereas clinical retro-auricular injection could not increase the efficiency of drug delivery into the ear. Electrocochleography results showed that Au@CD-PAMAM-Arg8/Dex significantly improved hearing in C57/BL6 mice after SM exposure. These findings indicate that Au@CD-PAMAM-Arg8 may be the useful drug carriers for the treatment of inner ear diseases.

## 1 Introduction

Approximately 250 million people worldwide suffer from disabling hearing loss and do not receive effective treatment (Holley, 2005). The main reason for treatment failure is the inability to deliver drugs effectively to the inner ear. Systemic intravenous administration, local tympanic injection, and surgery are the three most common modalities for treating ear diseases. The blood labyrinth barrier (BLB) filling with efflux pump proteins is an obstacle for reaching the hair cells or the spiral ganglion cells after systemic administration (Saito et al., 2001). It is difficult to deliver drugs through the round window membrane (RWM) to the inner ear via tympanic administration mainly because of the low expression of receptors on the RWM and the lipophilic nature of the cell membranes that prevents polar bioactive molecules such as proteins, peptides, and oligonucleotides from efficiently entering the inner ear (Kim, 2017; Zhang L. et al., 2018). Nanoparticles can overcome anatomical barriers and efficiently deliver encapsulated drugs to the target cells and organelles, and their usefulness in treating inner ear diseases remains to be fully exploited (Daraee et al., 2016; Lee et al., 2018).

**SCHEME 1:**
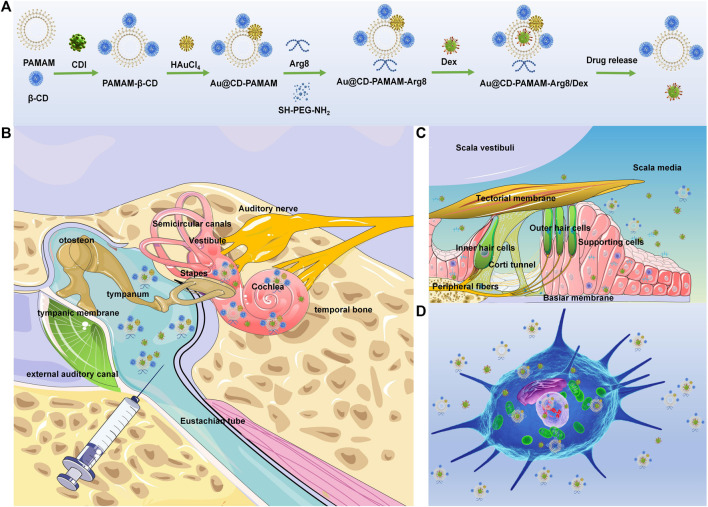
**(A)** Chemical synthesis of Au @PAMAM-β-CD-Arg8/Dex. **(B)** Retro-auricular RWM approach: the location of target cells (spiral ganglion, organ of Corti, and spiral ligament) and delivery route of Au @PAMAM-β-CD-Arg8/Dex nanoparticles in the inner ear through tympanic cavity injection. **(C)** OHCs are located on the basement membrane in scala media, with the scala vestibuli on top and the scala tympani below. Au @PAMAM-β-CD-Arg8/Dex targeted to hair cells, supporting cells, and other structures. **(D)** Schematic diagram of nanometers entering HEIOC1 cells.

By engineering nanoparticles with strong transmembrane penetration ability, biocompatibility, and sufficient cavity structure for drug loading, better drug delivery efficiency and specific cell targeting can be achieved (Scheme 1A). Poly amidoamine (PAMAM) dendrimers are widely used synthetic hyperbranched polymers with a high degree of monodispersity, polyvalency, and controlled molecular architecture. After modification, such as glycosylation and acylation, they have good biocompatibility and low toxicity (Markowicz et al., 2021). Compared to other common nanocarriers for drug-targeted delivery, such as solid lipid nanoparticles, micelles, and polymeric nanoparticles, PAMAM dendrimers are nonimmunogenic and have controlled molecular architecture and uniform particle size (Eichman et al., 2000). In addition, their internal cavity structure can improve the dispersibility of loaded drugs, and a large number of external end groups (-NH2, -COOH, and -OH surface groups) can be used to realize different targeting molecular ligands and fluorescent probe modifications (Parimi et al., 2010; Malinga-Drozd et al., 2021). Poly amidoamine (PAMAM) dendrimers can be modified with various groups with different functions (Tomalia et al., 1985; Tomalia et al., 1986), such as fluorophores, targeting ligands, and drugs, thereby expanding their applications to a variety of biological diagnostics and therapeutics (Shakhbazau et al., 2010; Labieniec-Watala and Watala, 2015). Third-generation poly amidoamine (PAMAM) dendrimers delivery systems have been developed for effective drug delivery to the inner ear. β-cyclodextrin (β-CD), considered safe by the US Federal Drug Administration (Crumling et al., 2012), is used to increase the biocompatibility of the PAMAM nano-system. The Au @CD-PAMAM system was developed by reducing HAuCl_4_ with PAMAM-β-CD. GNPs, surfaces of which are easily modified to deliver drugs, nucleic acids, and target tumors, have CT imaging capability and can reduce the cytotoxicity of vectors by peripheral modification of polyethylene glycol chains dendrimers (Nam et al., 2009; Hou et al., 2016) or by embedding into the internal cavity of dendrimers (Shan et al., 2012; Xiong et al., 2019) with remarkably strengthened cellular uptake and intracellular release efficiency (Hou et al., 2015). For better cellular internalization, Au @CD-PAMAM-Arg8 (Au-DENPs) have been synthesized by linking an oligoarginine peptide (Arg8) through the amide reaction (Maitani and Hattori, 2009). Peptides with low arginine levels can easily penetrate cell membranes as efficiently as other cell penetrating peptides (CPPs) and can enter the nucleus (Roy et al., 2010). Biofilms, such as RWM, have negatively charged surfaces; therefore, the uptake of drug delivery vectors into cells requires positively charged surfaces to bind to them. Cellular internalization achieved by macropinocytosis of oligoarginine peptide (Arg8) nanoparticles can improve drug penetration through the circular window membrane (Carlisle et al., 2001). The mechanism may be that binding to the cell surface through ion interactions between positively charged Arg8 and negatively charged surface proteoglycan induces megalocytosis (Nakase et al., 2004; Kitagishi et al., 2013).

Dexamethasone (Dex) can bind to glucocorticoid and mineralocorticoid receptors, which are widely expressed in the cochlea, thus preventing inflammation and maintaining lymphatic balance (Dinh et al., 2008; Meltser and Canlon, 2011). Several animal studies have shown that the intratympanic injection of free Dex can effectively alleviate SM ototoxicity (Kinis et al., 2013; Gül et al., 2017). Dex is also used to treat sudden sensorineural hearing loss, Meniere’s disease, and noise-induced hearing loss (Haynes et al., 2007; Stachler et al., 2012).

Therefore, in this study, we developed Dex-loaded nanoparticles for the effective delivery of Dex to the inner ear. A series of *in vitro* and *ex vivo* experiments were conducted (Scheme 1B–D). We also evaluated the biodistribution, cytotoxicity, and drug delivery efficiency of these nanoparticles (Scheme 1D) in an animal model (Scheme 1B, C).

## 2 Experimental Section

### 2.1 Materials

β-CD, Tween 80, 1-(3-dimethylaminopropyl)-3-ethylcarbondiimide hydrochloric acid (EDC) 98.5%, sodium borohydride 98%, N-hydroxy succinimide (NHS) 98%, and chloroauric acid 97% were purchased from Shanghai Macklin Biochemical Technology Co., Ltd. (Shanghai City, China). Dexamethasone was purchased from Sigma-Aldrich, Co. (Saint Louis, MO, United States). Arg8 peptides 98% were obtained from Hangzhou Dan gang Biotechnology Co., Ltd. (Hangzhou City, China). Dexamethasone fluorescein (Dex-FITC) was obtained from Invitrogen (Carlsbad, CA). Triton X-100 and dimethyl sulfoxide 99.0% (DMSO) were acquired from Wuhan Google Biotechnology Co., Ltd. (Wuhan City, China). Phosphate-buffered saline (PBS, pH 7.4) was purchased from Gibco (Grand Island, NY). The dialysis membrane (mol. wt. cut-off (MWCO): 1 kDa, 2 kDa) was purchased from Spectrum Laboratories (Rancho Dominguez, CA). Two-month-old C57/BL6 mice (30−40 g) were purchased from the Guangdong Experimental Animal Center. (Guangdong, China). All the animal experiments were conducted using protocols approved by the Experimental Animal Ethics Committee of the Rui ye Model Animal Center (Ethics Number: 20210111008).

### 2.2 Materials

#### 2.2.1 Description of PAMAM-β-CD

β-CD was used as a precursor for the nanoparticles. PAMAM-β-CD synthesis: β-CD (63.2 mg) and CDI (90.28 mg) were dissolved in DMSO (10 ml) with continuous stirring for 6 h, and the third-generation polyamide amine dendritic macromolecule (G3 PAMAM) (57.93 mg, 2.227 mol) dissolved in dimethyl sulfoxide (DMSO) was added dropwise to the solution with constant mechanical stirring (650 rpm) at room temperature for 3 days. The suspension was dialyzed against 0.01 M phosphate-buffered saline (PBS) using a 2-kDa dialysis bag for 24 h and purified using ultrapure water for 2 days to eliminate free chemical residues. The product was then freeze-dried, and the final white powder product (G3. NH_2_-β-CD) was stored at −20°C.

#### 2.2.2 Synthesis of Au @CD-PAMAM

Au @CD-PAMAM was produced by adding the G3. NH_2_-β-CD solution into an aqueous solution of chloroauric acid (HAuCl_4_) under continuous intense agitation for 30 min. Then, the aqueous solution of sodium borohydride (NaBH_4_) was quickly added five times to continue the reaction for 4 h. Subsequently, the copolymers were purified in distilled water using a 2-kDa dialysis bag for 72 h and then isolated by freeze-drying.

#### 2.2.3 Preparation of Nanocomposites With Drugs

Arg8 polypeptides (lipid concentration: 1.5 mg/ml), EDC (lipid concentration: 0.2 mg/ml), and NHS (lipid concentration: 0.4 mg/ml) were added into PBS (pH = 7.4) and stirred at room temperature for 4 h. The drug-loaded nanomaterial powder (Au@CD-PAMAM)was dissolved in PBS, and then the commixture of the two solutions was obtained and allowed to react overnight at roomtemperature. The mixture was freeze-dried to obtain the drug-loaded nanocompositematerial with targeted properties of Au @CD-PAMAM-Arg8(Au-DENPs). The free chemical residues were removed by dialysis using a 1-kDa MWCO membrane. Dexamethasone (Dex) dissolved in DMSO (concentration: 2 mg/ml) was added to the Au-DENPs. The mixed solution was stirred overnight at room temperature, filtrated with a 2-kDa MWCO membrane and freeze-dried to get Au @CD-PAMAM-Arg8/Dex (Au DENPs-Dex).

### 2.3 Material Characterization

#### 2.3.1 Characterization of Nanoparticle Carriers

The ^1^H NMR hydrogen spectrum of G3 NH_2_-β-CD was recorded using a Bruker AV Ⅲ 500 MHz NMR spectrometer (Wang et al., 2021). The FT-IR spectra of the G3. NH_2_-β-CD and Au @CD-PAMAM nanoparticles in the range of 4,000–500 cm^−1^ were examined by the potassium bromide tablet method. The morphology of Au @PAMAM-β-CD-Arg8 was determined by cold-emission, high-resolution transmission electron microscopy (TEM, JEM-2010F, JEOL., Ltd.). The size distribution and zeta potentials of the targeted drug-loaded nanocomposite suspensions were measured by the dynamic light scattering (DLS) technique using a Nano-ZS 90 Zetasizer (Malvern, America) at 25°C. The zeta potential (ζ) was determined by using laser Doppler electrophoresis (LDE) (Qiu et al., 2018; Wang et al., 2021). *In vitro* CT images of each concentration of the NPs (0.005, 0.01, 0.02, 0.04, 0.08, and 0.16 M) were captured using a single-source CT imaging system (Aquilion ONE 320 CT, Canon Medical Systems). All CT scans were performed at 100 kV voltage, 80 mA current, and a slice thickness of 0.625 nm. CT images were recorded, and the HU values of each sample were obtained by using built-in software.

#### 2.3.2 Drug-Loading Capacity and Drug Release *In Vitro*

The loading content (%) and loading efficiency (%) of Au-DENPs-Dex were measured by high-performance liquid chromatography [HPLC (Thermo Fisher U3000)] at following time points: 0.5, 1, 3, 5, 8, 24, 48, and 72 h (Yoon et al., 2015). The test conditions were as follows: a series of Dex solutions with different concentrations (1,000, 500, 250, 125, 62.5, 31.25, 15.6, 7.8, and 3.9 μg/m L), a mobile phase of water and acetonitrile (60:40 v/v), sample injection volume of 20 μL, flow rate of 1.0 ml/min, column temperature of 30°C, and detection wavelength of 240 nm (Zhao et al., 2021).

### 2.4 Cell Experiments

#### 2.4.1 Cell Culture

Although HEIOC1 cells were undifferentiated, both hair cell and support cell markers were expressed on them (Tan et al., 2020). The HEIOC1 cells were cultured in the high-glucose Dulbecco’s modified Eagle’s medium (high-glucose DMEM; Sigma-Aldrich, Saint Louis, United States) mixed with 10% fetal bovine serum (FBS; Gibco, BRL) and 1% penicillin-SM (Sigma, Saint Louis, United States). The cells were cultured in a humidified 10% CO_2_ environment at 37°C. The mouse fibroblasts L929 cells were maintained in the DMEM complete medium supplemented with 10% FBS and 1% penicillin-SM at 33°C in a humidified incubator with 10% CO_2_.

#### 2.4.2 Cytotoxicity

The cytotoxicity of Au @CD-PAMAM against HEIOC1 cells and L929 cells was quantitatively detected by the CCK-8 method. L929 cells and HEIOC1 cells at a density of 5,000 cells/well were inoculated into a 96-well plate and placed in a CO_2_ incubator for overnight culture and wall sticking. A medium with different concentrations of Au @CD-PAMAM (concentrations of Au @CD-PAMAM in HEIOC1 cells: 1, 5, 10, 50, 100, 200, 400, 600, and 800 ug/mL and concentrations of Au @CD-PAMAM in L929 cells: 50,100,200,400,600, and 800 ug/mL) and 0.01 M PBS was used as the control. Cells were detected by the CCK-8 method after culture for 24 h (Su et al., 2019). The microplate reader (Bio-Rad Laboratories, Hercules, CA) was used to determine the optical densities of the samples at 450 nm.

#### 2.4.3 Cellular Uptake

Cell uptake and intracellular localization of FITC-labeled Au-DENPs-Dex (0.6 mg/ml) in HEIOC1 cell were investigated after 0.5, 2, and 6 h of treatment using confocal microscopy (LSM880 Live Configuration Variotwo VRGB; Zeiss, Jena, Germany). The HEIOC1 cells were treated with FITC-labeled Au-DENPs-Dex at the maximum safe dose, determined by the cytotoxicity test. The cells were fixed with 4% paraformaldehyde (PA) solution after incubation and stained with DAPI (Sigma, Saint Louis, United States). A confocal microscope was used to observe the treated cells (Zambito et al., 2021).

#### 2.4.4 The Targeting of Nanoparticle Carriers

Cell uptake of the Au-DENPs complex with and without Arg8 functionalization was analyzed to confirm Arg8’s targeting ability. HEIOC1 cells were cultured overnight in a 96-well plate, and then FITC-labeled Au@PAMAM-β-CD-Arg8, FITC-labeled Au@PAMAM-β-CD (0.2 mg/ml), and 0.01 M PBS (pH = 7.4) were added and incubated for 0.5, 2, and 6 h. Cellular uptake efficiency in each group was quantified by flow cytometry. The untreated cells were used as negative controls. The corresponding fluorescence intensity (three parallel for each well) was quantified using Flow Jo 7.6.1 software.

### 2.5 Animal Experiments

#### 2.5.1 Nanoparticle Distribution in the Cochlea

Surgery: male C57/BL6 mice aged 2 months were anesthetized with intraperitoneal (I. p.) injection pentobarbitone (with 0.5% pentobarbital sodium at the dosage of 0.02 ml/g body weight) and placed on a temperature-controlled heating mat to hold body temperature at 37°C in the supine position. An incision along the posterior auricular sulcus was made, and the left-side of the otic vesicle was exposed, in which a small hole was made with an insulin syringe. Next, the maximum safe dose of Au-DENPs-Dex (0.4 mg/ml), FITC-labeled Au-DENPs (80 ug/mL), or free Dex (5 mg/ml) was injected into the tympanum until the cavity was filled. Bone wax was used to plug the hole after injection (JOHON, Shen Zheng City, China), and the wound was sutured. After surgery, baytril (1 mg/kg; Sigma-Aldrich, Saint Louis, MO, United States) was used to treat middle ear infection by intraperitoneal injection once daily (Yang et al., 2018).

After 0.5, 2, 3, 24, and 72 h of injection of Au-DENPs-Dex (0.4 mg/ml, 100 µL) and free Dex (5 mg/ml, 100 µL) into the middle ear cavity (*n* = 5/group, control group: *n* = 5), immunohistochemical staining with an antibody-targeting Dex was conducted. The ABC HRP Kit and a peroxidase substrate (Vector, Burlingame, CA) were used to detect nanoparticle distribution in the inner ear at different time points (Riquelme et al., 2010).

After 3 and 24 h of intratympanic injection and 3 h of tail intravenous injection of FITC-labeled Au-DENPs (0.01 M PBS was used as control), the cochlear basilar membranes of the group subjected to intratympanic injection for 3 h (*n* = 2/group) were incubated with Alexa Fluor 660-conjugated phalloidin to label actin, which is heavily expressed in the stereocilia and the cuticular plate of outer hair cells (OHCs), inner hair cells (IHCs), and pillar cells (PCs) and DAPI (Sigma-Aldrich, Saint Louis, United States). The other membranes were incubated with anti-β-tubulin Ⅲ antibody (Abcam, United Kingdom) to label never fibers (NFs) and spiral ganglion neurons (SGNs) and then immersed in a solution consisting of secondary antibody conjugated with Alexa Fluor 555 (1:200, Solarbio, Beijing City, China) normal goat serum, Triton X-100, and 0.01 M PBS as well as with DAPI. Then, the membranes were studied under a confocal microscope (Zeiss LSM-880) (Ding et al., 2011; Ding et al., 2012; Wang et al., 2017; Zhang J. et al., 2018) to determine the distribution of FITC-loaded nanoparticles in the inner ear after the two different injection methods.

#### 2.5.2 Nanoparticle Distribution *In Vivo*

A total of sixteen mice, aged 2 months, were injected with 100 μL of FITC-labeled Au-DENPs (2 mg/kg) into the tympanum, posterior ear, muscle, and tail vein. After 0.5, 1, 3, 5, and 7 h, fluorescent optical imaging was performed by using a small animal imaging system (FX Pro Bruker) to evaluate the enrichment of FITC-labeled Au-DENPs *in vivo* following the different injection methods. The mice were anesthetized and placed on the imaging stage. The excitation wavelength is 495 nm, and the emission wavelength was 519 nm. Acquisition and image analysis were performed using Living 302 Image 4.4.1 software.

The mice were injected with 100 μL of Au-DENPs (2 mg/kg) via the left tympanum and tail vein. The animals were euthanized after 5 h and the main organs (heart, liver, spleen, lung, kidney, and bilateral cochlear) were excised and subjected to induced coupled plasma atomic emission spectroscopy (ICP-AES) to explore the distribution of the nanoparticles *in vivo*.

#### 2.5.3 Toxicity Evaluation *In Vivo*

A total of twelve male C57/BL6 mice, aged 2 months, were divided into four groups (*n* = 3/group). The control group mice did not undergo surgery or treatment, and the other three groups received Au-DENPs-Dex, Au-DENPs, or Au @CD-PAMAM treatment via caudal vein injection (0.6 mg/ml; 100 uL). The mice were euthanized 2 weeks later, and H&E staining was conducted to observe the cytotoxicity of the nanoparticles in the main organs (heart, liver, spleen, lung, and kidney).

### 2.6 Drug Delivery Efficiency of the Nanoparticles in the SM Ototoxicity Animal Model

#### 2.6.1 Cytotoxicity and Therapeutic Effects in Cells

The HEIOC1 cells were treated with the fresh complete medium with various concentrations of SM (0, 1, 2, 5, 10, 15, 20, 30, and 40 mg/ml) to determine the optimal dosing concentration of SM. In another 96-well plate, the HEIOC1 cells were dealt with the best dosing concentration of SM after pretreatment with six different concentrations (1, 5, 10, 15, 20, and 30 μg/ml) of Au-DENPs-Dex and Dex alone; 0.01 M PBS was used as the control group (Zhao et al., 2021). Then, the cytotoxicity of SM to HEIOC1 and the efficacy of free Dex and Au-DENPs-Dex in treating SM ototoxicity were quantitatively detected by the CCK-8 method to verify drug delivery efficiency of nanoparticles.

#### 2.6.2 Cytotoxicity and Therapeutic Effects in Animals

Compared with auditory brainstem response (ABR) *in vivo*, compound action potential (CAP) in electrocochleography (ECochG) was good in reflecting the functional status of the peripheral auditory neurons and cochlear sensory epithelium following SM damage. The mice were anesthetized 3 days after tympanic cavity injection of Au-DENPs-Dex mixed with SM, mixture of SM and Dex, SM (concentration of Au-DENPs-Dex or Dex: 30 μg/ml; SM: 37.5 mg/ml, 100 µL), or 0.01 M PBS. After exposing the left facial nerve to set a silver wire into the facial nerve canal for CAP recording, the reference electrode and ground electrode were inserted subcutaneously at the earlobe of the test ear and the opposite earlobe, respectively (Li et al., 2021). The data were collected by using an analyzer (Neurosoft LLC, Russia). ECochG click was generated in response to tones (1, 2, 3, 4, 6, 8, 16, and 32 kHz) between 100 and 20 dB in 5 dB increments.

After CAP recording, the mice were euthanized, and the basement membranes were stained with anti-β-tubulin Ⅲ antibody, secondary antibody conjugated with Alexa Fluor 488, Alexa Fluor 660-conjugated phalloidin, and DAPI to evaluate the damage caused to hair cells, spiral ganglion cells, nerve fibers behind the inner hair cells, and ganglion cells following treatment of SM, Au-DENPs-Dex, and Dex alone (Ding et al., 2011; Zhang J. et al., 2018).

## 3 Results and Discussion

### 3.1 Properties of Nanoparticle Carriers

**FIGURE 1:**
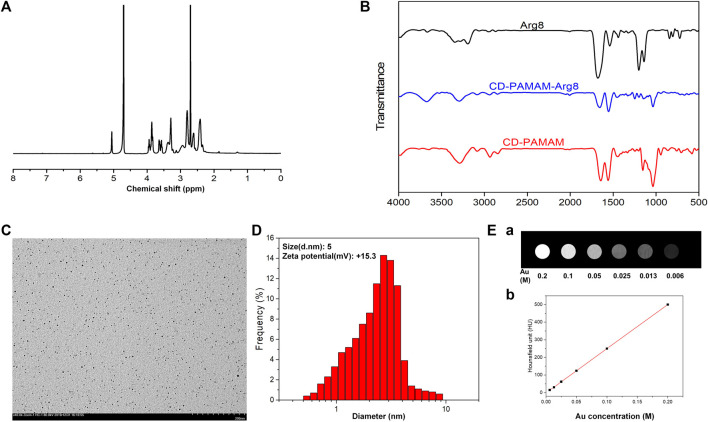
**(A)** ^1^H NMR spectrum of G_3_-β-CD (β-CD). **(B)** FT-IR spectra of Arg8, Au@CD-PAMAM-Arg8, and Au @CD-PAMAM. **(C)** TEM image and **(D)** particle size distribution of Au@CD-PAMAM-Arg8. **(E)** Au @CD-PAMAM CT images of different concentrations.

The characteristic ^1^H NMR peaks of the G3 PAMAM dendrimer and β-CD in the presence of G3-β-CD are shown in Figure 1A. The chemical shift between 2.25 and 2.34 ppm is due to the methylene skeleton of the G3 PAMAM dendrimer, whereas the proton peaks around 3.5–4 and 5 ppm are due to the β-CD group. The FT-IR spectra of the prepared samples, Arg8, Au-DENPs, and Au @CD-PAMAM are shown in Figure 1B. The IR-Vis spectrum of Arg8 showed a single deep Soret band ranging from 1,750 to 1,650 nm, indicating intermolecular self-aggregation. With a concomitant increase in the new signals upon the addition of Au @CD-PAMAM, the single deep Soret band reduced. The λ max of the Soret band (1,320–1,250 nm) and a broad and deep band (800–630 nm) in the final spectra was in good agreement with that of the inclusion complex of Au @CD-PAMAM. Upon the addition of Au-DENPs to the solution, the double Soret band ranging from 1,750 to 1,650 nm gradually declined (Kermanian et al., 2021).

The transmission electron microscopy (TEM) image of G3-β-CD is shown in Figure 1C. The prepared Au @CD-PAMAM was small with an average size of about 2.9 nm and was spherical shaped. It showed good dispersion in aqueous solutions, with uniform particle size distribution. Some research studies considered that the shape and size of the drug-carrier particle greatly influence the ability to pass into the organ of Corti and uptake into cells. Nanoparticles less than 200 nm can effectively penetrate through the RWM (Kim et al., 2015). The size of our nanoparticles, characterized by dynamic light scattering, was uniform and small, averaging about 5 nm (Figure 1D), consistent with the TEM results. Biofilms, such as RWM, have negatively charged surfaces, which can bind with a positively charged Arg8 to increase the uptake into hair cells (Zhang L. et al., 2018). Zeta potential measurements revealed that our samples of Au-DENPs nanoparticles were positively charged (+15.3 mV).

As shown in Figure 1E, the CT image gradually brightened with the increase in Au concentration. When Au concentration was 0.1 m, the luminance of the CT image was obviously enhanced. Compared with the clinical use of Omnipaque, Au @CD-PAMAM had a high HU value at the same concentration, suggesting that Au @CD-PAMAM has a potential clinical value as a CT imaging contrast agent.

### 3.2 Drug Loading and Release

**FIGURE 2:**
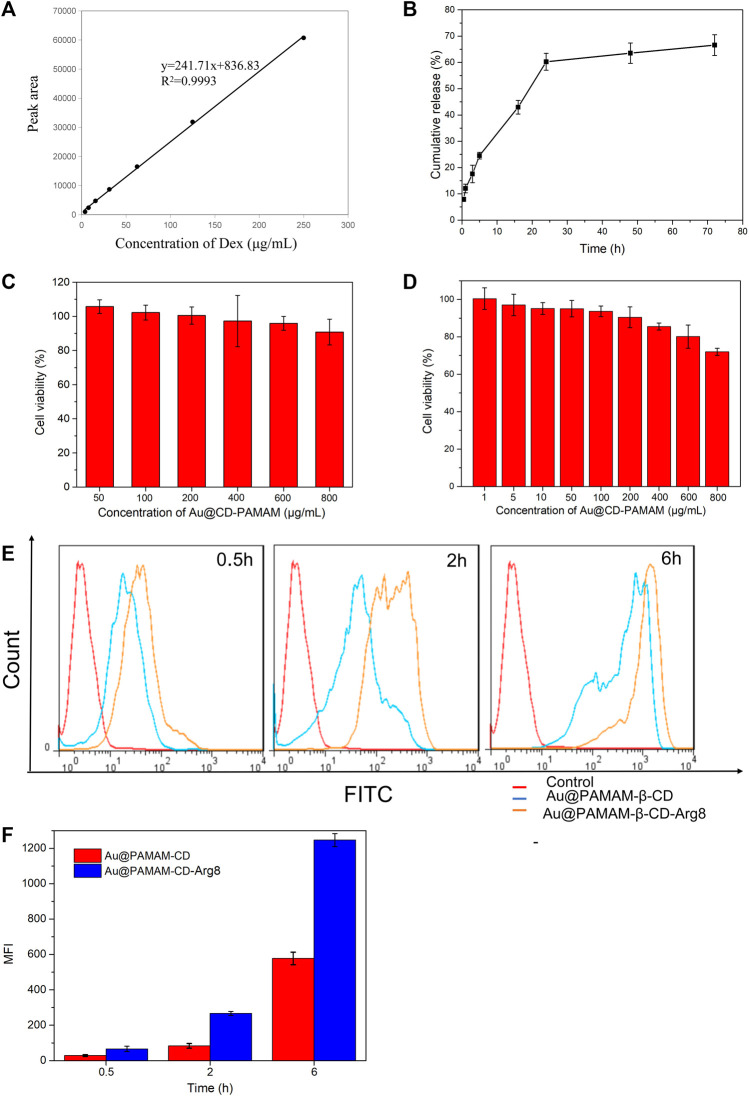
**(A)** Standard curve of Dex. **(B)** Release curve of Dex. **(C)** L929 cell cytotoxicity. **(D)** HEIOC1 cell cytotoxicity. **(E)** Targeting of nanoparticle carriers. **(F)** Quantitative analysis of gold nanoparticles expression in HEIOC1 cells at different time intervals.

The relationship between different concentrations of Au-DENPs-Dex solution and peak area was measured by HPLC. The standard curve equation Y = 241.71× +836.83, R2 = 0.9993 was obtained, and the drug loading was 1.3% (Figure 2A). Dex was loaded onto Au-DENPs. The particle diameter was about 5 nm, which was similar to that before drug loading. The loading efficacy was 93.1 ± 7.6% (1.3%). According to recent studies, Dex remains in the outer lymphatic fluid of the cochlea for not more than 12 h after a single tympanum administration (Sun et al., 2015). However, our nanoparticles remained in the dendritic structure for a longer period owing to hydrophobic interactions. Burst release was observed at 24 h, after which the release rate decreased. After 70 h, the release rate of Dex was 70%, indicating that our nanoparticles have long-lasting effects (Figure 2B).

### 3.3 Cytotoxicity and Absorption Rates of Nanoparticle Carriers

Au @CD-PAMAM showed mild toxicity at 0.8 mg/ml in L929 cells (Figure 2C) and at 0.4 mg/ml in HEIOC1 cells (Figure 2D). Cellular internalization achieved by oligoarginine peptide (Arg8) nanoparticles can improve drug penetration through the circular window membrane into the cells of the inner ear and even into their nuclei, thus delivering the drugs to the specific target. The results of our flow cytometry analysis confirm this. The relative fluorescence intensity of Au-DENPs (Au@CD-PAMAM-Arg8) was enhanced to a great extent compared to that of the Au @CD-PAMAM nanoparticles, and the uptake increased exponentially from 0.5 to 6 h (Figures 2E,F). These findings indicate that Arg8 can increase the transmembrane ability of nanoparticles.

### 3.4 Cellular Uptake

**FIGURE 3:**
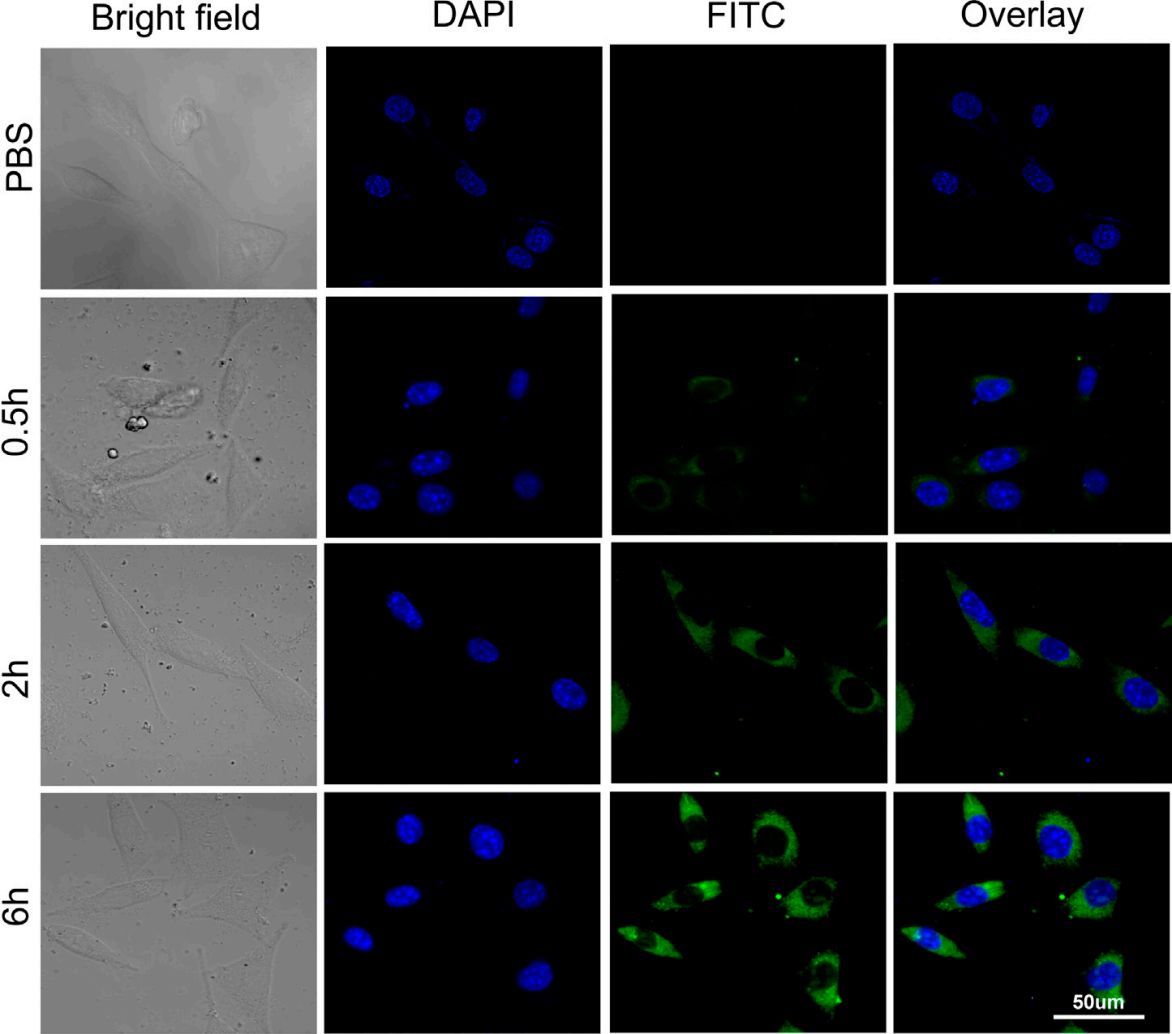
Cell uptake of FITC-labeled Au-DENPs in HEIOC1 cells at 0.5, 2, and 6 h.

The results of the confocal microscopy studies were in line with those of flow cytology analysis. After 0.5 h of treatment with FITC-labeled Au-DENPs (0.4 mg/ml) nanocarriers, green FITC fluorescence was detected at the periphery of the cell nuclei stained with DAPI. The staining intensity gradually increased with time, and the fluorescence could be clearly observed in the cells and the nuclei after 6 h. These findings confirm that Arg8-conjugated nanoparticles can penetrate the cell membrane and reach the nuclei (Figure 3).

### 3.5 Nanoparticle Distribution in the Cochlea

**FIGURE 4:**
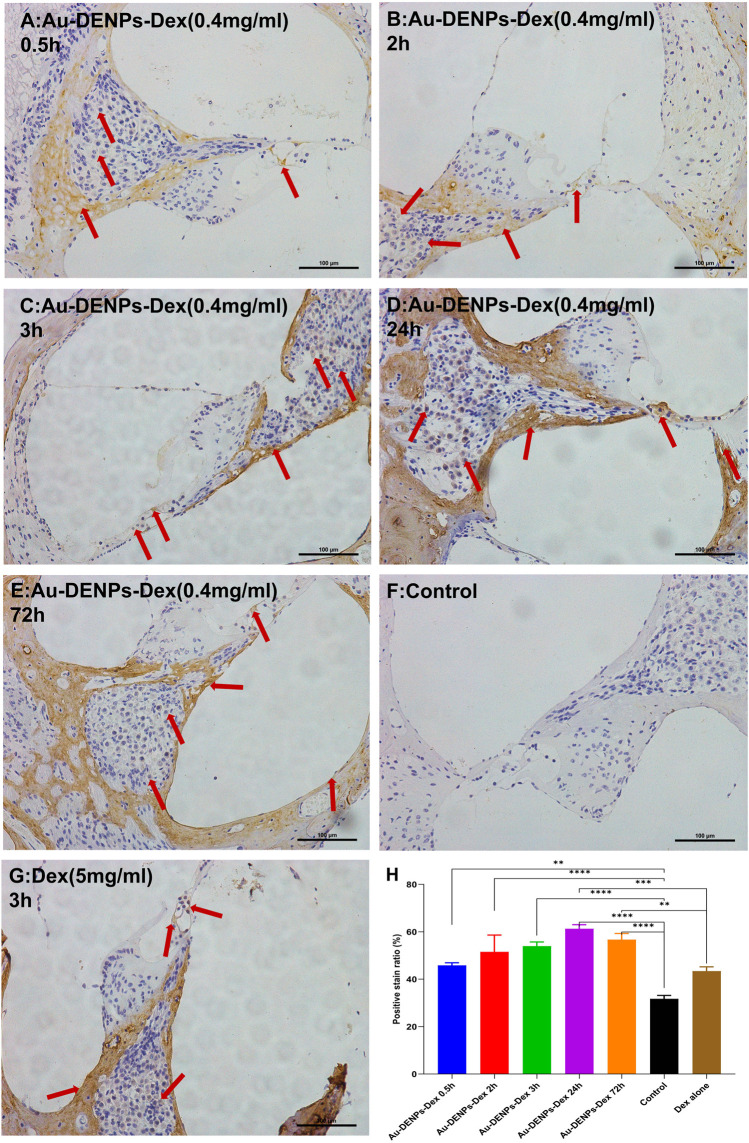
Immunostaining for dexamethasone (Dex) after injection of Au-DENPs-Dex, free Dex, and PBS. **(A–E)** Tympanum injection of Au-DENPs-Dex (400 ug/ml) for 0.5, 2, 3, 24, and 72 h, respectively. **(F)** Tympanum injection of 0.01 M PBS for the control group. **(G)** Tympanum injection of free Dex (5 mg/ml) for 3 h as a positive group. **(H)** positive staining rate of each group. *p < 0.05, **p < 0.01, ***p < 0.001, and ****p < 0.0001.

Most drugs, such as steroids, used in inner-ear disease are hydrophobic and not conducive to cellular internalization. However, Au-DENPs-Dex showed uptake of Dex on the spiral ganglion cells, the modiolus, the medial side, hair cells, and the lateral wall after 0.5 h of treatment; this increased markedly after 24 and 72 h (Figures 4A,D,E). Moreover, in our study, the positive staining rate of the group treated for 3 h with Au-DENPs-Dex (400 ug/mL) was significantly higher than that of the group treated with Dex alone (5 mg/ml) (Figures 4C,F). This confirmed that Arg8 and β-CD-conjugated nanoparticles are likely to improve absorption of hydrophobic drugs by cells and facilitate rapid action of these drugs. As Dex is encapsulated in nanoparticles, the distribution of Dex indirectly reflects the distribution of the nanoparticles. Nanoparticles began to appear around the spiral ganglion cells, modiolus, and medial side 0.5 h after administration and were present in the vicinity of OHCs and IHCs after 2 h, as shown in Figure 4B. However, according to recent research, in parts of the inner ear, such as Reissner’s membrane, the organ of Corti, and the stria vascularis, tight junctions (TJs) directly exerted profound effects on intercellular sealing for complete perilymph and endolymph compartmentalization and prevented leakage of solutes through a paracellular pathway (Kitajiri et al., 2004). Therefore, we conjectured that absorption through the modiolus or limbus and diffusion to the hair cells were the main routes through which nanoparticles in the perilymph approached the hair cells, instead of through the tight junctions in the superior margin of the intercellular space of the organ of Corti, which is linked with the endolymph.

**FIGURE 5:**
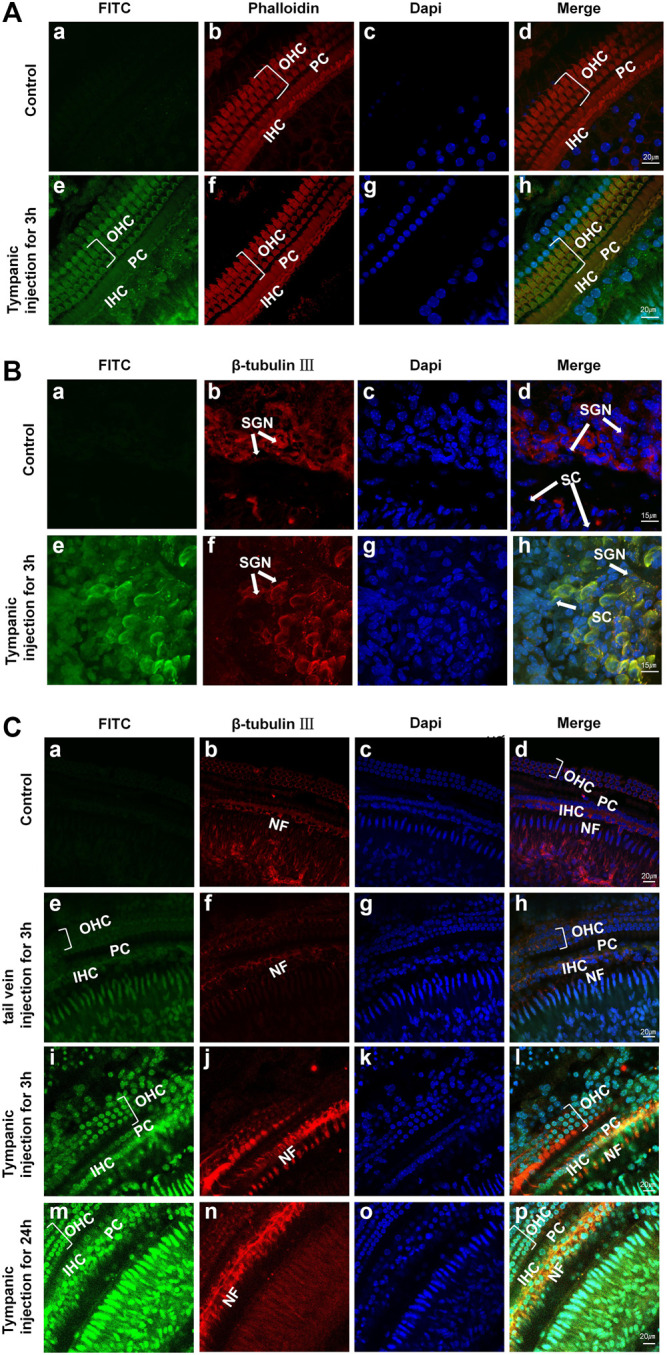
**(A,B)** Confocal microscopy images of OHCs, IHCs, and SGNs in adult mice after 3 h of PBS (a–d: control group) and FITC-labeled Au-DENPs treatment (e–h) by tympanum injection (*n* = 2 per group). **(C)** Confocal microscopy images of the organ of Corti in adult mice after nanoparticles delivery by tail vein injection for 3 h and tympanum injection for 3 and 24 h and control group (*n* = 2 per group). Green represents FITC-labeled Au-DENPs nanoparticles **(A,C),** and red represents OHCs and IHCs stained with phalloidin in (A) or SGN and NF stained with β-tubulin Ⅲ in **(B,C)**. DAPI was used to label nuclei **(A–C)**.

Studies have shown that SM ototoxicity is mainly manifested in the nerve endings associated with the auditory receptors, OHCs, IHCs, and SGN (Wang et al., 2017; Ding et al., 1993; Cui et al., 2015). Hence, it is greatly important to administer a large drug dose in OHCs, IHCs, nerve fibers, and SGN; however, drugs or genes in the perilymph have more difficulty reaching the organ of Corti than reaching the spiral ligament or the spiral ganglion (Ding et al., 2014). Our confocal microscopy images of the cochlea delivery of FITC-labeled Au-DENPs (80 ug/mL, 100 uL) showed that FITC uptake mainly appeared in OHCs and IHCs especially in NF and SGN in adult mice after tympanum injection (Figures 5A–C) and tail intravenous injection (Figure 5C, e). The fluorescence was the strongest for tympanic injection in the 24-h group (Figure 5C m) and the weakest for tail vein injection in the 3-h group. Furthermore, the fluorescence of the spiral ganglion was significantly stronger than that of the hair cells 3 h after intratympanic injection (Figures 5A,B). Both the hair cells and SGN were not damaged after exposure. Moreover, a recent anatomical study of the human cochlea revealed that perforated structures at the modiolar surface of the scala tympani (ST) and the scala vestibuli (SV) allowed fluid exchanges between the modiolar space/vascular tree, the perilymph, and the spiral ligament (Rask-Andersen et al., 2006). Our nanoparticles, with a diameter of 5 nm, are much smaller than those used in other experiments (diameter >200 nm) (Zhao et al., 2021). These results suggest that the size of nanoparticles affects absorption, and drugs with a small size in the perilymph reach the modiolus or limbus more easily. This further demonstrates the potential of our nanoparticles in the management of inner-ear disease.

### 3.6 Nanoparticle Distribution *In Vivo* With Different Injection Methods

**FIGURE 6:**
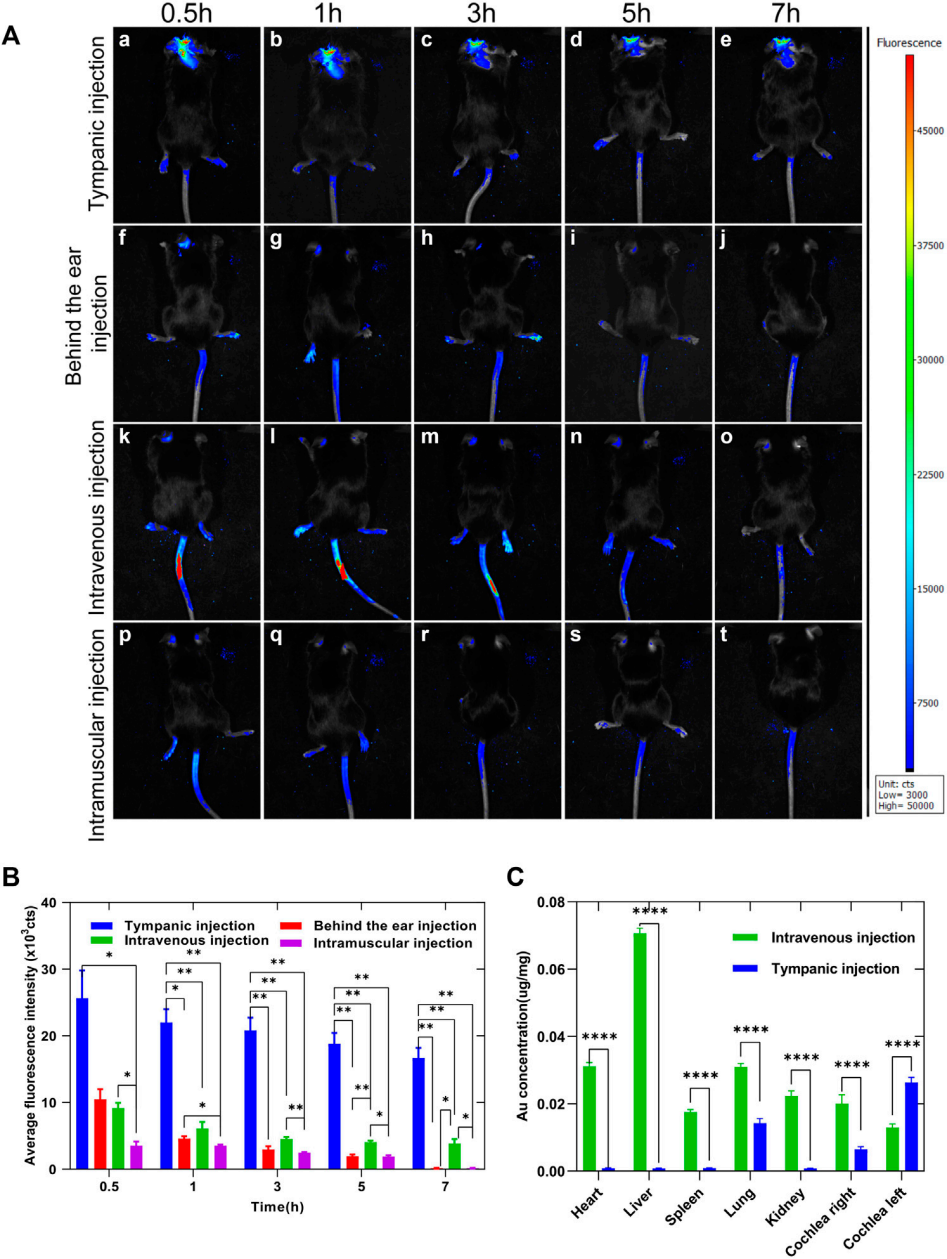
**(A)** Enrichment of Au-DENPs in the cochlea by different injection methods after 0.5, 1, 3, 5, and 7 h using a small animal imaging system. **(B)** Quantitative fluorescence of temporal bone after injection in tympanum, posterior auricular sulcus, vein, and muscle. **(C)** Gold content in the main organs after 5 h of tympanum and intravenous injection of Au-DENPs (2 mg/kg) with ICP-AES.*p < 0.05, **p < 0.01, ***p < 0.001, and ****p < 0.0001.

Fluorescent optical imaging was performed on mice to evaluate the enrichment and metabolism of Au-DENPs in the cochlea by different injection methods at different time periods. Accumulation of the fluorescent tracer reflected the number of nanoparticles entering the inner ear when various injection methods were used. The nanoparticles entering the inner ear reached maximal levels at 0.5 h (Figure 6) and gradually declined after 7 h (Figure 6A e, j, o, t and Figure 6B). The intratympanic injection route afforded the most intense fluorescent signals of FITC, followed by the intravenous, intramuscular, and retro-auricular sulcus injection routes (in that order). The results were analyzed by quantitative analysis of cochlea imaging data, and the results of quantitative analysis were significantly consistent with those obtained from *in vivo* imaging (Figure 6B). Many doctors choose posterior ear injection in clinics to increase the efficiency of drugs in the inner ear; however, we found that the amount of Au-DENPs entering the vessels of the inner ear by retro-auricular injection was similar to that by intramuscular injection (Figure 6A, f, p), and this basically confirmed that the effects of intramuscular injection were similar to those of retro-auricular sulcus injection in clinics. Fluorescent optical imaging only showed nanocarriers in the capillaries of the inner ear. While this did not mean that the nanocarriers crossed the blood–labyrinth barrier (BLB) and entered the epithelial cells of the inner ear labyrinth and the inner ear, Figure 5 C (a-d and e-h) shows that FITC fluorescence could be observed in the spiral ganglion cells and hair cells of the intravenous injection group, indicating that our nanoparticles could enter the inner ear through the BLB, and they had the potential to target focus of diseases in the inner ear.

### 3.7 Nanoparticle Distribution in Visceral Organs With the ICP-AES Test

ICP-AES was used to measure the distribution of gold elements in important organs. As shown in Figure 6C, the absolute gold content decreased in all organs. However, after left tympanum injection, Au-DENPs were relatively enriched in the left cochlea and lung and relatively low in other organs. In contrast, after intravenous injection, Au-DENPs were mainly enriched in the liver, heart, and lung, and low in the cochlea, with no observable difference between the left and right cochlea. This can be seen from ICP-AES results shown in Figure 6C. Therefore, tympanic injection of nanomedicine is an appropriate way to treat diseases of the inner ear (Figure 6C).

### 3.8 Toxicity Evaluation of Nanoparticles

**FIGURE 7:**
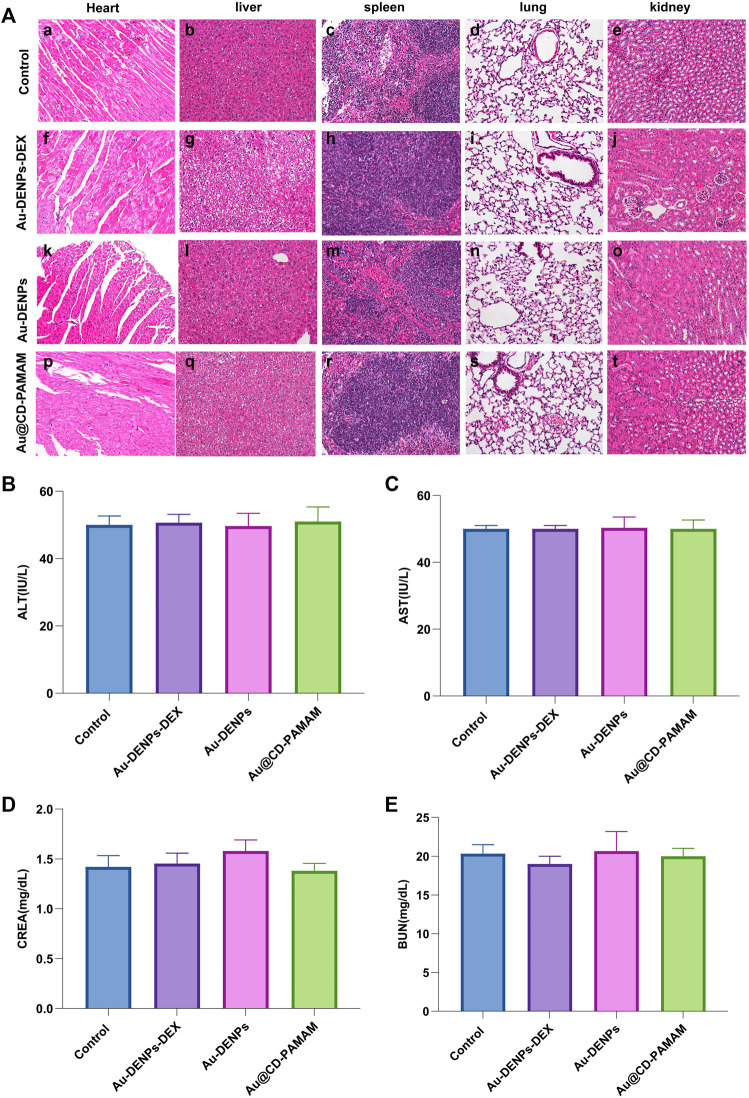
**(A)** Systemic biosafety assessment. H&E staining of the heart, liver, spleen, lung, and kidney of mice from each group 2 weeks after treatment. Scale bar = 100 μm. Assessment of acute toxicity effects of **(B)** ALT, **(C)** AST, **(D)** CREA, and **(E)** BUN of each group (*n* = 3 per group).

H&E staining was used to evaluate the systemic biosafety of Au-DENPs-Dex *in vivo* 2 weeks after treatment. Compared to the control group, all treatment groups (Au-DENPs-Dex group, Au-DENPs group, Au @CD-PAMAM group, or control group) had no obvious morphological changes or tissue damage (Figure 7A). The liver functionality (AST/ALT) (Figures 7B,C) and renal functionality (CREA/BUN) (Figures 7D,E) in each group was within the normal range, indicating that the nanoparticles exerted no obvious biological toxicity.

### 3.9 Drug Delivery Efficiency of Nanoparticles by the SM Ototoxicity Model

#### 3.9.1 Assessment of Therapeutic Effect

**FIGURE 8:**
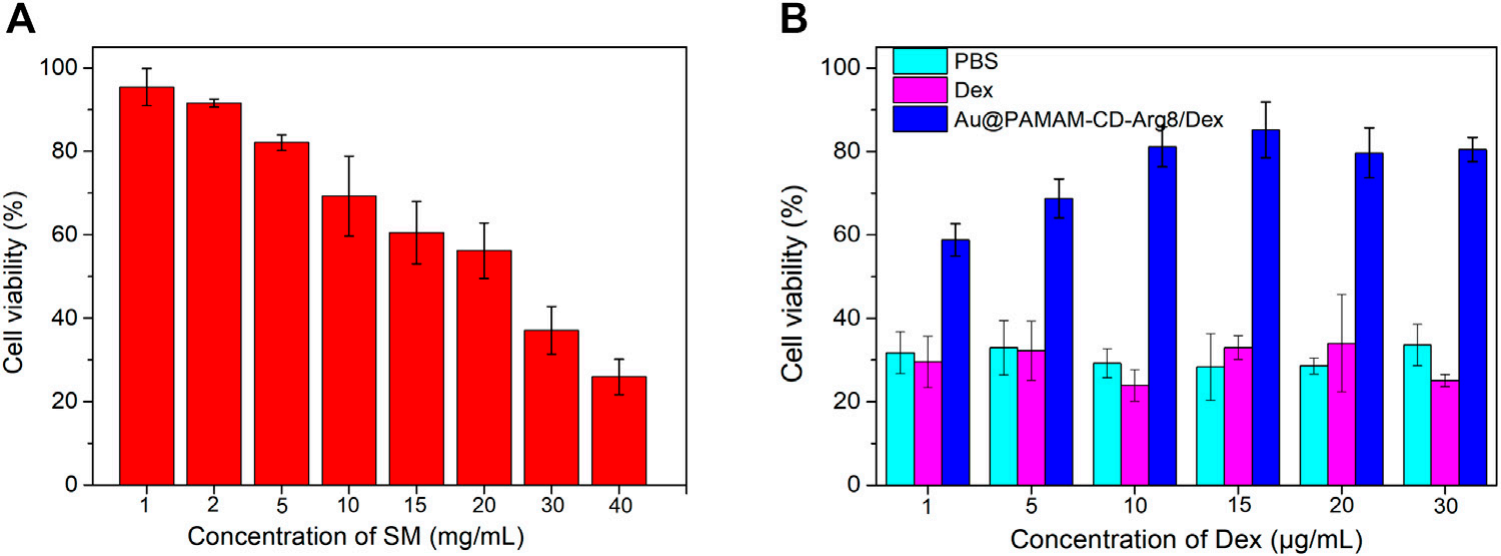
**(A)** Ototoxicity induced by SM at different concentrations. **(B)** Therapeutic effects of Dex alone and Au@CD-PAMAM-Arg8/Dex on streptomycin ototoxicity.

As shown in Figure 8A, an increase in SM concentration led to a gradual decrease in cell activity. An SM concentration of 30 mg/ml was used as an ototoxicity model to explore the therapeutic effects of treatments using Au-DENPs-Dex and dexamethasone only. As the concentration of Au-DENPs-Dex nanoparticles accumulated, the cell viability gradually increased. The cell viability of the Au-DENPs-Dex nanoparticles group, at the same concentration, was higher than that of both Dex only and 0.01 M PBS groups (Figure 8B). It indicated that the therapeutic effect of our nanoparticles is superior to that of direct drug administration at the cellular level.

#### 3.9.2 Auditory Function Evaluation *In Vivo*

**FIGURE 9:**
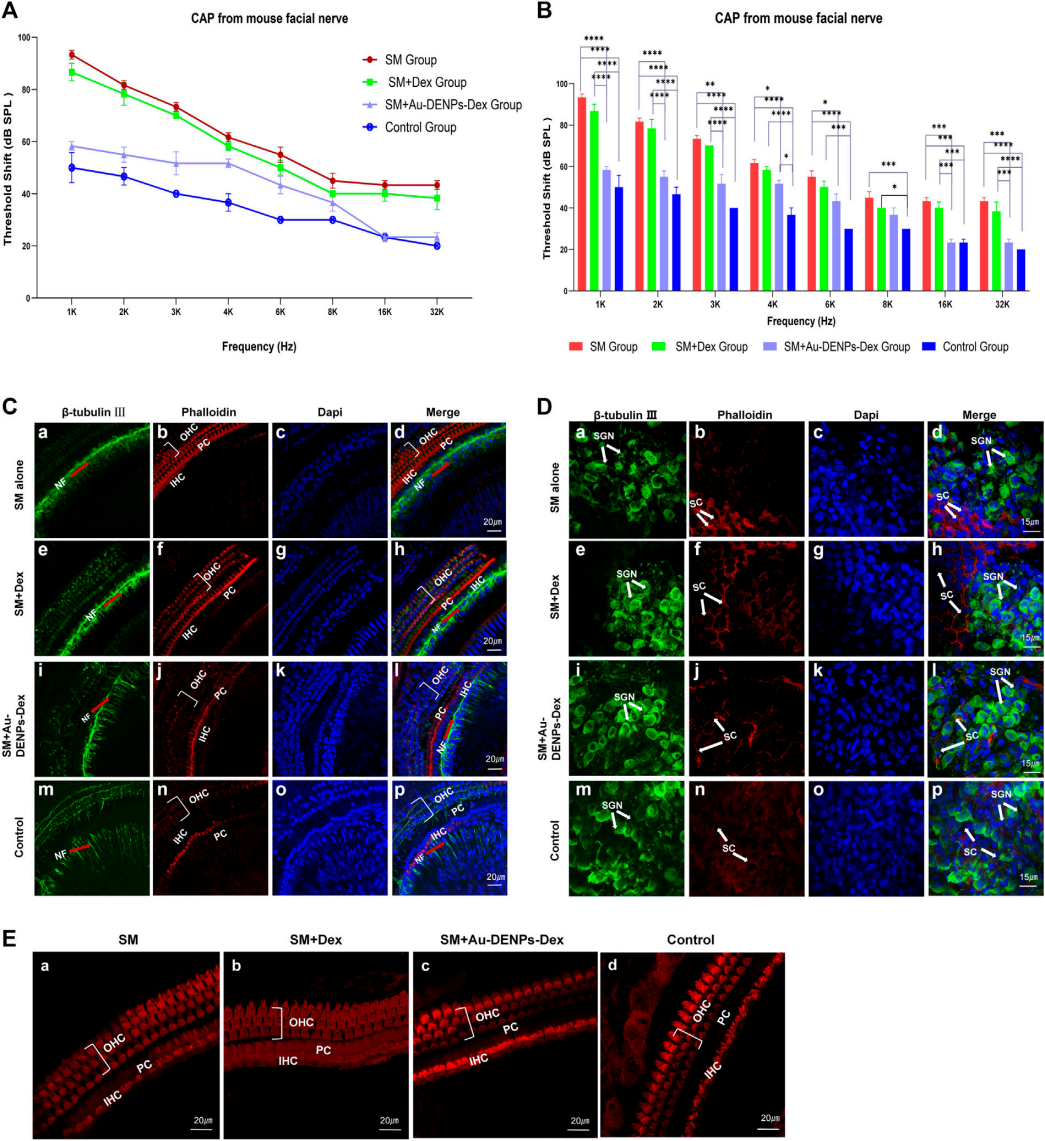
**(A)** Rapid changes in the CAP threshold of mice recorded from the facial nerve after treatment with streptomycin alone, streptomycin mixed with Dex, and streptomycin mixed with Au-DENPs-Dex (Au@CD-PAMAM-Arg8/Dex). **(B)** Comparison of hearing improvement among each group in response to 1,2,3,4, 6, 8, 16, and 32 kHz; *p < 0.05, **p < 0.01, ***p < 0.001, and ****p < 0.0001. The damage of nerve endings below the inner hair cells **(C)**, spiral ganglion cells **(D)**, and OHCs and IHCs **(E)** with different treatment methods under confocal microscopy.

After SM treatment, overproduction of ROS contributed to the production of pro-inflammatory cytokines, which severely exacerbated inflammation in the cochlea (Ding et al., 1993; Wrzesniok et al., 2013; Cui et al., 2015). The administration of Dex can reduce pro-inflammatory cytokines (Shan et al., 2012; Xiong et al., 2019). To further explore the drug delivery efficiency of nanoparticles, CAP thresholds recorded from the facial nerve for 1, 2, 3, 4, 6, 8, 16, and 32 kHz were performed 3 days after surgery, to investigate hearing function *in vivo*. There was a significant effect from SM alone, SM mixed with Dex, SM mixed with Au-DENPs-Dex treatment, and control groups on CAP thresholds [F (3, 64) = 188.7, *p* < 0.0001]. Hearing losses in the SM and SM mixed with Dex groups were significant compared to the control and SM mixed with Au-DENPs-Dex groups. (Figures 9A,B). Almost no hearing loss was reported in the SM mixed with Au-DENPs-Dex group. CAP thresholds in the SM group (ranging from 93.3 dB SPL at 1 kHz to 43.3 dB at 32 kHz) increased more than those in the SM mixed with Au-DENPs-Dex group (ranging 58.3 dB SPL at 1 kHz to 23.3 dB SPL at 32 kHz). This shows that Au-DENPs-Dex is effective for reducing SM-induced injury. The therapeutic effect of nanoparticles is greater than that of direct administration.

#### 3.9.3 Assessment of Therapeutic Effects *In Vivo*

Confocal microscopy images of nerve endings below the inner hair cells (Figure 9C), spiral ganglion cells (Figure 9D), and hair cells (Figure 9E) were used to explore the therapeutic effect of Au-DENPs-Dex and Dex on SM ototoxicity. In the SM mixed with Au-DENPs-Dex and control groups, a thick bundle of NF can be seen running medial and parallel to the row of IHC (Figures 9C l,p), and thin fascicles of NF project out radially from the thick fiber bundle in the direction of the OHC. Many blue-labeled nuclei were observed in the outer sulcus cell (OSC) area radial to the OHC (Figures 9C l,p), while the longitudinal NF bundle was medial to the row of IHC in the SM group.

The longitudinally oriented fascicle of NF medial to the IHC in the SM (Figure 9C, a) and SM mixed with Dex groups (Figure 9C, e) was severely damaged and the thickness was less than that in the control (Figure 9C, m) and SM mixed with Au-DENPs-Dex groups (Figure 9C i). SGNs were described as large, round, blue-labeled nuclei surrounded with the green cytoplasm marked with β-tubulin Ⅲ; numerous diminutive blue-labeled nuclei appeared in the circumambient support cells labeled with phalloidin (Figures 9D a,e,i,m). However, mostly β-tubulin-labeled SGNs were eliminated (Figures 9D d,h), and the soma and nuclei were shrunken in the SM or SM mixed with Dex groups (Figures 9D d,h) compared to the control and SM mixed with Au-DENPs-Dex groups (Figures 9D p,l). In addition, there was no significant difference in hair cells of each group; stereocilia and hair cells in all frequency regions of the organ of Corti in each group exhibited almost no damage and appeared similar to those of the control (Figure 9E).

We observed an apparent recovery in the morphology of nerve fibers, nerve endings below the inner hair cell, and SGN in the SM mixed with Au-DENPs-Dex group compared to the SM and SM mixed with free Dex groups (Figure 9), supporting the concept that acute cochlear damage caused by low concentrations of streptomycin mainly occurred in the nerve fibers, nerve endings below the inner hair cell, and SGN. Destruction of the efferent endings by SM leads to an increase in the auditory threshold. It is evident that Au-DENPs-Dex is significant for SM ototoxicity. These results indicate that the systemic toxicity of nanomaterials is negligible and has a potential clinical value in SM-induced ototoxic injury or other inner ear disease.

## 4 Conclusion

In summary, we successfully developed a safe and highly effective nano-drug carrier based on β-CD and Arg8-modified gold nanoparticles, Au-DENPs, targeted to inner ear diseases, which can be loaded with Dex and has promising treatment effects over Dex alone. We also verified that Au-DENPs-Dex is mainly distributed in SGN, hair cells, and supporting cells in the cochlea, which are major damage sites in cases of tinnitus and deafness. *In vivo* tracer tests showed that the effect of tympanic injection was significantly better than that of posterior ear injection, muscle injection, and tail vein injection, and clinical retro-auricular injection cannot increase the efficiency of administering drugs into the ear. The SM ototoxicity model verified the efficiency and potential of drug carriers for loading drugs *in vivo*. This research suggests that Au-DENPs are a potential new drug carrier, and the clinical application of Au-DENPs-Dex in acute inner ear injury is promising.

## Data Availability

The original contributions presented in the study are included in the article/Supplementary Material; further inquiries can be directed to the corresponding author.
