# Corrigendum: β-Cyclodextrin and Oligoarginine Peptide-Based Dendrimer-Entrapped Gold Nanoparticles for Improving Drug Delivery to the Inner Ear

**DOI:** 10.3389/fbioe.2022.921652

**Published:** 2022-05-23

**Authors:** Jia Luo, XueXin Lin, LiLing Li, JingQian Tan, Peng Li

**Affiliations:** ^1^ Department of Otolaryngology Head and Neck Surgery, The Third Affiliated Hospital of Sun Yat-Sen University, Guangzhou, China; ^2^ Department of Otolaryngology Head and Neck Surgery, The Eighth Affiliated Hospital of Sun Yat-Sen University, Shenzhen, China

**Keywords:** drug delivery, dexamethasone, inner ear, spiral ganglion, outer hair cells, nerve fibers

In the original article, there was a mistake in Figure 9E, panel d as published. Figure 9E has been redrawn and the corrected Figure 9E appears below.

**FIGURE 9:**
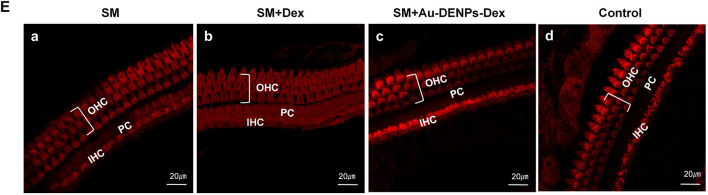
**(A)** Rapid changes in the CAP threshold of mice recorded from the facial nerve after treatment with streptomycin alone, streptomycin mixed with Dex, and streptomycin mixed with Au-DENPs-Dex (Au@CD-PAMAM-Arg8/Dex). **(B)** Comparison of hearing improvement among each group in response to 1, 2, 3, 4, 6, 8, 16, and 32 kHz; *p < 0.05, **p < 0.01, ***p < 0.001, and ****p < 0.0001. The damage of nerve endings below the inner hair cells **(C)**, spiral ganglion cells **(D)**, and OHCs and IHCs **(E)** with different treatment methods under confocal microscopy.

In the original article, there was an error in section two, **Experimental Section**, whereby several descriptions for the preparation of nanocomposites CD-PAMAM-Arg8 and CD-PAMAM-Arg8/Dex were omitted.

The following correction should be made to **Experimental Section**, “2.2.3 Preparation of Nanocomposites With Drugs”, paragraph 1:

“Arg8 polypeptides (lipid concentration: 1.5 mg/ml), EDC (lipid concentration: 0.2 mg/ml), and NHS (lipid concentration: 0.4 mg/ml) were added into PBS (pH = 7.4) and stirred at room temperature for 4 h. The drug-loaded nanomaterial powder (Au @CD-PAMAM) was dissolved in PBS, and then the commixture of the two solutions was obtained and allowed to react overnight at room temperature. The mixture was freeze-dried to obtain the drug-loaded nanocomposite material with targeted properties of Au @CD-PAMAM-Arg8(Au-DENPs). The free chemical residues were removed by dialysis using a 1-kDa MWCO membrane. Dexamethasone (Dex) dissolved in DMSO (concentration: 2 mg/ml) was added to the Au-DENPs. The mixed solution was stirred overnight at room temperature, filtrated with a 2-kDa MWCO membrane and freeze-dried to get Au @CD-PAMAM-Arg8/Dex (Au DENPs-Dex).”

The authors apologize for this error and state that this does not change the scientific conclusions of the article in any way. The original article has been updated.

